# Efficacy of vitamin C as an adjunct to fluoxetine therapy in pediatric major depressive disorder: a randomized, double-blind, placebo-controlled pilot study

**DOI:** 10.1186/1475-2891-12-31

**Published:** 2013-03-09

**Authors:** Mostafa Amr, Ahmed El-Mogy, Tarek Shams, Karen Vieira, Shaheen E Lakhan

**Affiliations:** 1 Department of Psychiatry, Mansoura University, Mansoura, Egypt; 2 Biosciences Department, Global Neuroscience Initiative Foundation, Los Angeles, California, USA; 3 Neurological Institute, Cleveland Clinic, Cleveland, Ohio, USA; 4 Department of Intensive Care, Mansoura University, Mansoura, Egypt

## Abstract

**Background:**

Current antidepressants used to treat pediatric patients have the disadvantage of limited efficacy and potentially serious side effects. The purpose of this study was to assess the efficacy of vitamin C as an adjuvant agent in the treatment of pediatric major depressive disorder in a six-month, double-blind, placebo-controlled pilot trial.

**Methods:**

The study group (n=12) was given fluoxetine (10–20 mg/day) plus vitamin C (1000 mg/day) and control group (n=12) administered fluoxetine (10–20 mg/day) plus placebo. The data were analyzed by ANOVA and *t*-test for independent samples.

**Results:**

Both groups demonstrated significantly improved scores on the Children’s Depression Rating Scale (CDRS), the Children’s Depression Inventory (CDI), and the Clinical Global Impression (CGI). ANOVA was significantly different on all clinical measurements (group effect, time effect, and interaction), with the exception of group effect and interaction for CGI. Patients treated for six months with fluoxetine and vitamin C showed a significant decrease in depressive symptoms in comparison to the fluoxetine plus placebo group as measured by the CDRS (*t*=11.36, *P*<0.0001) and CDI (*t*=12.27, *P*<0.0001), but not CGI (*t*=0.13, *P*=0.90). No serious adverse effects were observed.

**Conclusions:**

These preliminary results suggest that vitamin C may be an effective adjuvant agent in the treatment of MDD in pediatric patients.

## Introduction

The prevalence of depression in community settings has been estimated to be between 0.4% and 2.5% in children and 0.4% and 8.3% in adolescents [1]. However, in a more recent community study of children without depression who were initially assessed between the ages of 9 and 13 years, more than 7% of boys and almost 12% of girls developed a depressive disorder by the age of 16 [2].

While the diagnosis of major depressive disorder (MDD) in younger patients generally follows the criteria set forth in the Diagnostic and Statistical Manual of Mental Disorders, 4th edition, text revision (DSM-IV-TR) [3], the treatment of pediatric depression presents many challenges. Not only do children with MDD have multiple co-morbid disorders [4,5], psychosocial and academic problems, and are at increased risk for suicide attempts, self-harm, and substance abuse [1,6-10], treatment options are often limited and ineffective, poorly tolerated, and generally present long delays in delivering a therapeutic benefit [11-14].

One of the few antidepressants approved for use in children is the selective serotonin reuptake inhibitor (SSRI) fluoxetine [15]. The first study that demonstrated the positive effects of using fluoxetine to treat depression in child and adolescent patients was published in 1997 [16], following a small trial in which no difference was observed between fluoxetine treatment and placebo [17]. Overall, five clinical trials have been conducted which show the positive effects of using fluoxetine to treat pediatric depression [16,18-21]. In addition, the improvement response rate on the Clinical Global Impressions (CGI) for antidepressant use was found to be between 52% and 61% for fluoxetine patients versus 33% to 37% for patients treated with placebo [22]. The CGI measures whether or not depressive symptoms have improved after treatment.

Despite being one of the most popular treatments for pediatric patients, in 2004 the use of prescription medication such as fluoxetine as well as other antidepressant medications declined by approximately 20% in the United States [23]. This shift in prescription patterns is likely due to warnings issued by regulatory agencies, initially in the United Kingdom [24] and later in the United States [25], against the use of SSRIs to treat depression in pediatric populations due to the possible link between antidepressant usage and an increased incidence of suicidal ideations or attempts. Subsequently, there is a compelling need for better understanding of the pathophysiology of MDD as well as the development of novel treatment methods that can be used to improve the current clinical management of pediatric depression.

Nutrients like vitamin C (ascorbic acid) have become of interest in adjuvant therapy settings for the management of depressive symptoms due to the fact that psychological abnormalities are among the characteristics of vitamin C deficiency [26-29]. A recent population-based survey revealed that 60% of the patients in the acute medical wards of a Montreal teaching hospital were vitamin C deficient, while this deficiency was only detected in 16% of people attending the hospital’s outpatient center [30]. There is also preliminary evidence that the administration of vitamin C may be able to reduce the severity of MDD in both children [31] and adults [32], as well as improve mood in healthy individuals [33-35]. In addition, a recent study reported a 35% reduction in average mood disturbance in hospitalized patients following treatment with vitamin C (1000 mg/day) [36]. In one particular study that investigated mood, patients who were acutely hospitalized were either treated with vitamin C or vitamin D as a deficiency in both of these vitamins has been associated with psychological abnormalities [32]. The results showed that only vitamin C led to an improved mood. More specifically, treating the vitamin C deficiency led to a decrease in mood disturbance while vitamin D supplementation had no effect on mood. Similar findings were observed in non-critically ill hospitalized patients who were treated with vitamin C for hypovitaminosis C [36]. Moreover, an animal study showed that the co-administration of vitamin C was found to potentiate the action of subeffective doses of fluoxetine (1 mg/kg) [37]. This synergistic antidepressant effect of vitamin C and fluoxetine suggests that this vitamin could be helpful in improving conventional pharmacotherapy for pediatric MDD and potentially reduce side effects.

This study would be the first to examine the efficacy of vitamin C as an adjunct to SSRIs in the treatment of pediatric depression. In addition, the low potential toxicity, inexpensiveness, and over-the-counter availability, we sought to investigate whether oral supplementation of vitamin C would improve clinical depressive symptoms. Therefore, the present study was designed to measure the effect of vitamin C on the Children’s Depression Rating Scale (CDRS), the Children’s Depression Inventory (CDI), and the CGI scores in pediatric patients with depression taking fluoxetine.

## Methods

### Trial design

The study was a prospective, double-blind, placebo-controlled, six-month clinical trial. Two parallel groups of outpatient pediatric patients with depression in Mansoura University Hospital, Egypt participated in the study from October 2009 to September 2011. The study was approved by the institution’s review board.

### Participants

The authors screened pediatric patients (less than 18 years of age) who were referred to the outpatient psychiatry clinic for MDD based on a semi-structured interview and DSM-IV-TR criteria [3]. Exclusion criteria included clinically significant organic or neurological disorder, psychotic disorder or depression with psychotic features, a history of substance abuse or dependence, or prior use of psychotropic medication. Young patients with bipolar disorder may experience adverse psychological effects such as mania and hypomania due to antidepressants and were therefore, excluded from the study. It has been shown that patients who are young in age at the onset of bipolar disorder demonstrate an illness progression that is characterized by high rates of switching into mania or hypomania in response to antidepressant treatment [38]. Among the 32 patients screened during this period, five were excluded (two had depression with psychotic features, two had a history of hypomania, and one had a substance abuse disorder). The remaining 27 patients agreed to participate in this study after informed consent from at least one parent was obtained. The patients did not receive any other treatment such as cognitive behavioral therapy during the trial period. This trial was performed in accordance with the Declaration of Helsinki and subsequent revisions [39]. Written consent was obtained from each patient’s parent or guardian before entering the study.

### Intervention

Vitamin C and placebo were formulated into capsules by the Mansoura University Hospital. The patients were randomly allocated to either the treatment or control group using a computer-generated list of random numbers. Fourteen patients were assigned to the treatment group and were given fluoxetine (10–20 mg/day) plus vitamin C (1000 mg/day; 500 mg BID). Thirteen patients were assigned to the control group and were given fluoxetine (10–20 mg/day) plus placebo. Patients less than eight years of age received fluoxetine (10 mg/day), whereas patients eight years of age or older were given 10 mg/day of fluoxetine for one week and 20 mg/day all subsequent weeks as per the prescribing information [40]. There are several published studies which support the administration of 20 mg/day of fluoxetine for children at least eight years of age [16,18,20,21], and it is within FDA indication. The use of fluoxetine for children under the age of eight is off-label. A dose of 1000 mg/day of vitamin C (500 mg BID) was chosen based on human studies suggest that psychiatric patients generally require higher levels of vitamin C to improve symptoms than the doses that are recommended for healthy individuals [32,41]. The recommended dose of vitamin C for healthy individuals is 70 mg/day, while a dose of 1000 mg/day needs to be consumed before symptoms begin to improve in psychiatric patients [41].

Patients in the placebo group received two identical capsules (morning and evening). No other psychotropic medications were prescribed. Three subjects were removed from the trial due to noncompliance (two patients from the vitamin C group and one from the placebo group). Patients were assessed using CDRS, an Arabic version of CDI, and CGI at the baseline as well as 3 and 6 months after the start of treatment. The scores for the CDRS were based on parent ratings, CDI on children ratings, and CGI on clinician ratings. Examinations of patients during the treatment period were performed by a psychiatrist trained in the use of these instruments.

### Instruments

The Children’s Depression Rating Scale (CDRS) is a 16-item measure used to determine the severity of depression in children and adolescents aged 6 to 12 [42]. The CDRS is derived from the Hamilton Rating Scale for Depression (HAM-D) [43] and is based on parent, child, and schoolteacher interviews. CDRS scores show good concordance with research diagnostic criteria for depression [44] and correlate highly with other interview and self-report measures of depression severity [45].

The Children’s Depression Inventory (CDI) is a 27-item, self-rated, symptom-oriented scale suitable for children and adolescents aged 7 to 17 [46]. The CDI is sensitive to changes in depressive symptoms over time, making it a useful index for the severity of MDD. The CDI is reported to have high internal consistency and test-retest reliability [47]. The CDI assessment utilized in this study was based on the previously developed instrument [46] and was translated and normalized for Arab children by Gharib (1988) [48]. Reliability and validity data for the Arabic version are comparable to those provided for the original instrument.

The Clinical Global Impressions Scale (CGI) is a 3-item, observer-rated scale that measures illness severity, global improvement or change, and therapeutic response [49]. The CGI is rated on a 7-point scale with each component being rated separately; the instrument does not yield a global score. Over the past 30 years, the CGI has been shown to correlate well with standard, well-known research drug efficacy scales, including the Hamilton Rating Scale for Depression, the Brief Psychiatric Rating Scale, and the Scale for the Assessment of Negative Symptoms across a wide range of psychiatric indications [50].

### Statistical analysis

Student’s *t*-tests and chi-squared tests were used to evaluate possible differences in baseline demographics. Two-way repeated measures analysis of variance (ANOVA) were used to assess the effects of treatment (treatment versus placebo), time (months of visit), and an interaction between the treatment and time. Significant differences in the mean scores for each visit were assessed through unpaired Student’s *t*-tests. Quantitative variables were tested for normal distributions by the Kolmogorov-Smirnov test. The variables were presented as means ± standard deviations (SD). Statistical significance was set at the 5% level. SPSS for Windows version 13 was used for the statistical analysis of the data obtained from the study.

## Results

### Demographic characteristics and attrition

**Figure 1:**
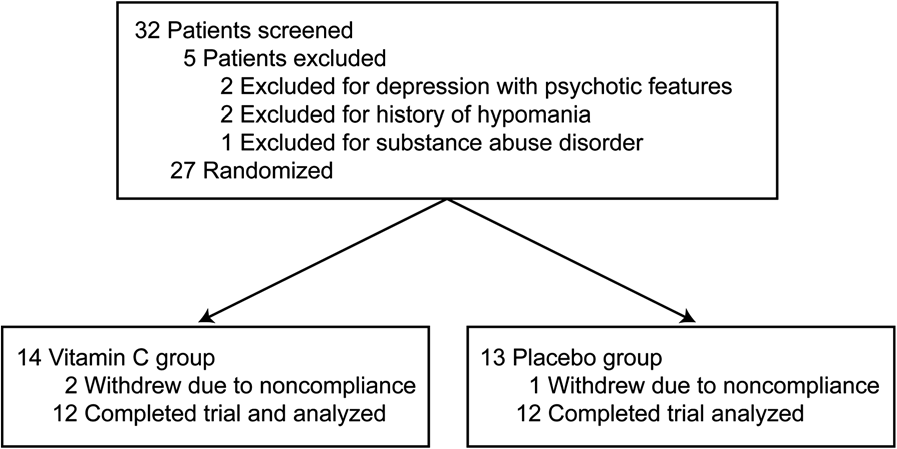
Diagram demonstrating the disposition of all patients screened for the study.

Thirty-two patients were initially examined, but five patients did not satisfy the inclusion criteria. Therefore 27 patients enrolled in the study; 14 assigned to the vitamin C group and 13 to the placebo group. Twenty-four patients aged between 7 and 14 completed the six-month trial. Two patients from the vitamin C group and one patient from the placebo group were removed from the trial due to noncompliance (Figure 1).

The characteristics of the two study groups are summarized in Table 1. The two groups were well matched, and there were no statistically significant differences between the groups with regard to demographic factors or duration of illness.

**Table 1:** Demographic data of the participants in fluoxetine + vitamin C and fluoxetine + placebo groups

**Characteristic**	**Vitamin C group**	**Placebo group**	***P***
**Age (years)**	10.3 **±**2.2	9.9 **±**2.1	0.653
**Sex (M/F)**	7/5	8/4	0.673
**Duration of illness (months)**	4.3 **±**1.1	4.5 **±**0.1	0.5370

Statistical significance defined as *P*<0.05.

### Effect on CDRS, CDI and CGI scores

**Figure 2:**
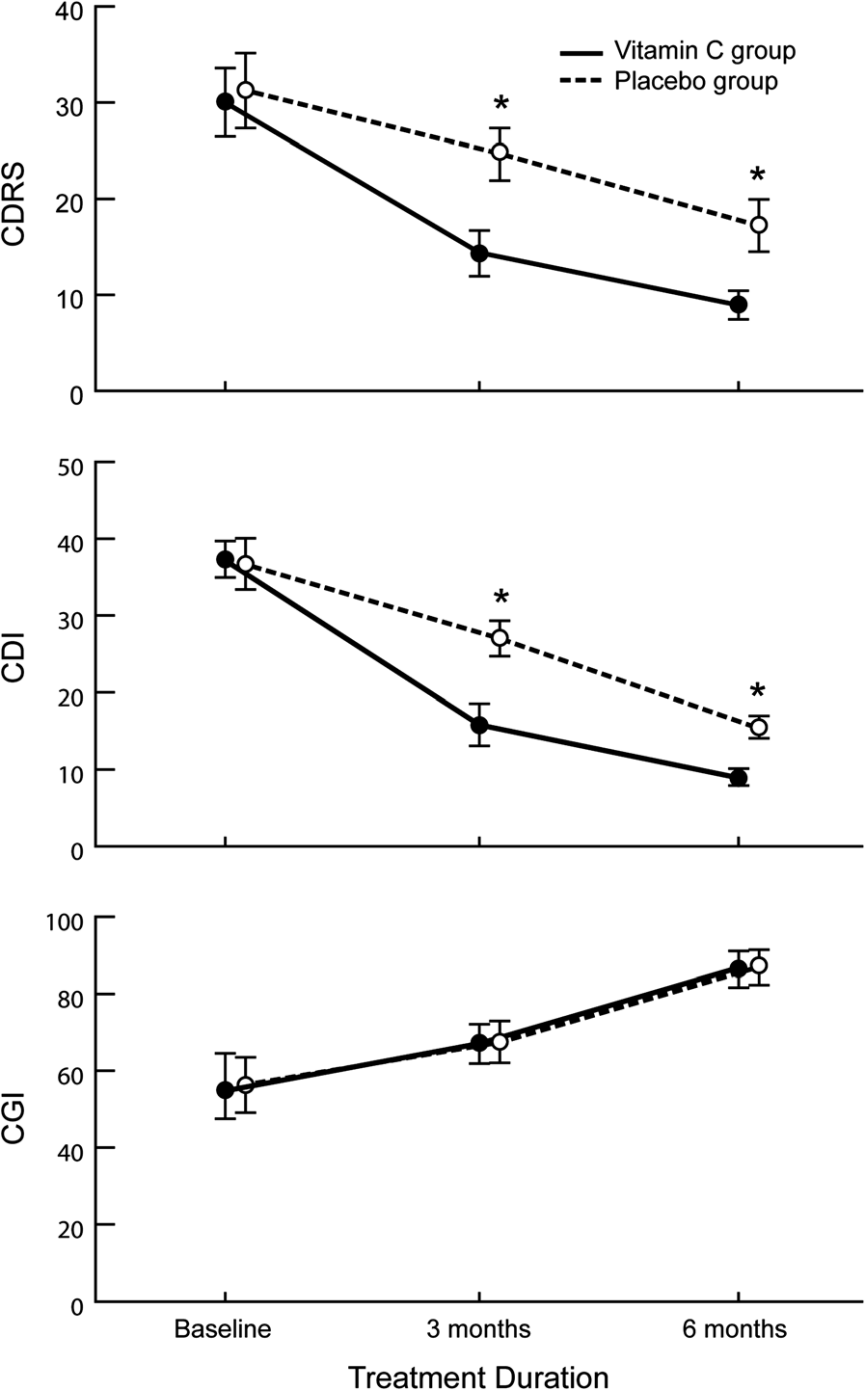
**Mean and SD changes in CDRS, CDI, and CGI scores at baseline and after 3 and 6 months in fluoxetine + vitamin C and fluoxetine + placebo groups.** Asterisk indicates statistical significance defined as *P*<0.05. CDI: Children’s Depression Inventory; CDRS: Children’s Depression Rating Scale; CGI: Clinical Global Impression; SD: standard deviation.

The clinical severity of the depression was comparable at baseline and not significantly different across all three clinical instruments. As shown in Figure 2, mean scores for CDRS, CDI, and CGI gradually improved in both study groups during the trial. ANOVA was significantly different on all clinical measurements (group effect, time effect, and interaction), with the exception of group effect and interaction for CGI. The results of ANOVA are presented in Table 2.

**Table 2:** Repeated-measures analysis of variance assessments at baseline and after 3 and 6 months in fluoxetine + vitamin C and fluoxetine + placebo groups

	**Group effect**			**Time effect**			**Interaction**		
**Variable**	***F***	***Df***	***P***	***F***	***Df***	***P***	***F***	***Df***	***P***
**CDRS**	155.90	1, 11	<0.0001	294.59	1.6, 17	<0.0001	25.14	1.4, 18.4	<0.0001
**CDI**	89.69	1, 11	<0.0001	512.77	1.8, 19.4	<0.0001	57.21	1.8, 19.8	<0.0001
**CGI**	0.000	1, 11	1.000	447.95	1.6, 17.2	<0.0001	0.68	1.1, 12.1	0.438

Statistical significance defined as *P*<0.05. CDI: Children’s Depression Inventory; CDRS: Children’s Depression Rating Scale; CGI: Clinical Global Impression.

The most striking effects were observed for the interaction between treatment and time: CDRS (*F*=25.16, *df*=1.4, 18.4, *P*<0.0001) and CDI (*F*=57.21, *df*=1.8, 19.8, *P*<0.0001). There was no significant interaction effect in CGI scores (*F*=0.68, *df*=1.1, 12.1, *P*=0.438).

The clinical changes demonstrated with vitamin C intake compared to placebo were noted by the third month of the study (Table 3). At three months, a significant difference was observed on CDRS (*t*=9.85, *P*<0.0001) and CDI (*t*=10.77, *P*<0.0001), but not CGI (*t*=0.15, *P*=0.88). By the end of trial (six months), the vitamin C group showed a significantly larger decrease in depressive symptoms compared to the placebo group as measured by CDRS (*t*=11.36, *P*<0.0001) and CDI (*t*=12.27, *P*<0.0001). However, the effect on CGI was not significantly different (*t*=0.13, *P*=0.90) at six months.

**Table 3:** CDRS, CDI, and CGI scores at baseline and after 3 and 6 months in fluoxetine + vitamin C and fluoxetine + placebo groups

**Variable**	**Treatment duration**	**Vitamin C group**	**Placebo group**	***t***	***P***
**CDRS**	Baseline	30.1 **±**3.56	31.3 **±**3.89	0.77	0.45
	3 months	14.4 **±**2.39	24.7 **±**2.73	9.85	<0.0001
	6 months	9.0 **±**1.50	17.3 **±**2.73	11.36	<0.0001
**CDI**	Baseline	37.3 **±**2.38	36.7 **±**3.33	−0.42	0.68
	3 months	15.8 **±**2.75	27.0 **±**2.30	10.77	<0.0001
	6 months	9.0 **±**1.12	15.5 **±**1.45	12.27	<0.0001
**CGI**	Baseline	54.7 **±**7.19	56.3 **±**7.23	0.57	0.58
	3 months	67.1 **±**5.04	67.4 **±**5.55	0.15	0.88
	6 months	86.5 **±**4.91	86.7 **±**4.41	0.13	0.90

Values given are mean ± standard deviation. The statistics listed were measured with *t*-test. Statistical significance defined as *P*<0.05. CDI: Children’s Depression Inventory; CDRS: Children’s Depression Rating Scale; CGI: Clinical Global Impression.

### Clinical complications and adverse effects

No major adverse effects were observed.

## Discussion

These results show that orally administered vitamin C as an adjunct to fluoxetine treatment leads to significantly greater decreases in depressive symptoms in comparison to fluoxetine treatment alone. This was demonstrated by the decrease in depressive symptoms, which was observed in the improved CDRS and CDI scores. A significant effect was not observed for the CGI, but this may be related to the response items for this instrument. For instance, symptoms were scored according to whether “much improvement” or “very much improvement” was observed [49]. Although there may have been a slight increase in CGI scores, response items such as these may have made it difficult to detect a significant improvement of symptoms. The differences between the scores may have also been related to the individuals who supplied the ratings for each instrument. More specifically, the scores for the CDRS were based on parent ratings, the scores for the CDI were based on children ratings, and the scores for the CGI were based on clinician ratings. The scores from the CGI were computed based on clinical criteria such as that which is listed in the DSM-IV-TR as well as semi-structured interviews. Therefore, the clinician’s rating and score interpretations adhered to strict guidelines and training, whereas the ratings from parents and children may have been more subjective leading to significantly different scores. Nonetheless, these preliminary findings, including the results of ANOVA suggest that vitamin C may be an effective adjuvant agent for the treatment of depression in pediatric patients. Furthermore, the results support the notion that vitamin C has antidepressant-like properties and are in accordance with previous animal research that demonstrated vitamin C’s ability to potentiate the action of conventional antidepressants [37].

Despite the lack of research investigating the effects of vitamin C in pediatric patients with MDD, previous studies have suggested that vitamin C improves clinical symptoms in other psychiatric disorders [51-53], and that vitamin C supplementation can be used to positively modulate mood [33-35]. Furthermore, Khanzode et al., (2003) showed that plasma levels of vitamin C were decreased in depressive patients [54]. In a more recent study, Chang et al., (2007) described a case in which a patient with depression developed scurvy, suggesting that reduced plasma levels of vitamin C due to inadequate vitamin C intake could be associated with the pathophysiology of depression [29]. Other studies have also shown that depressive symptoms are associated with scurvy [55-57].

While the exact role of vitamin C in the etiology of MDD is not well understood, a growing body of evidence suggests that oxidative stress, characterized by an accumulation of free radicals due to an organism’s inhibited antioxidant capacity, may play a primary or secondary role in the pathogenesis of neurological and psychiatric diseases like MDD [58,59]. The brain is much more vulnerable to oxidative free radicals than other tissues since it utilizes 20% of the oxygen consumed by the body, contains large amounts of polyunsaturated fatty acids and iron, and typically has low concentrations of antioxidant enzymes [60]. Previous studies have shown that MDD may be accompanied by disturbances in the balance between pro- and anti-oxidative processes, demonstrated by decreased blood plasma levels of the antioxidants enzymes superoxide dismutase, catalase, and glutathione peroxidase and an increased level of lipid peroxidation by-products in patients with depression versus healthy controls [54,61,62].

While antidepressant drugs may affect the oxidative or antioxidative systems [54], partly due to their effects on the immune [63] and P450 systems [64], adjunctive therapy with vitamin C may provide additional protection as it is the brain’s most abundant antioxidant and plays an important role in preventing free radical-induced damage [65,66]. In addition to its neuroprotective properties, vitamin C has also been identified as a neuromodulator in the brain, modulating both dopamine- and glutamate-mediated neurotransmission [67-69]. As there is a considerable amount of pharmacological evidence demonstrating the efficacy of antidepressants with dopaminergic effects in the treatment of depression [70], vitamin C’s complex interaction with the dopaminergic system may be another potential mechanism of action. However this effect appears to be dose-dependent. Wambebe and Sokomba (1986) showed that administering 50–200 mg/kg of vitamin C to rats enhanced dopamine-mediated behavioral effects [71], while higher dosages have been shown to antagonize such effects [68].

There are a number of other potential biological substrates that underlie vitamin C’s effects on depression and mood. For example, Binfaré et al. [37] identified the involvement of 5-HT_1A_ receptors in the antidepressant-like effect of vitamin C. Additionally, adjuvant administration of vitamin C may also prove useful in decreasing the risk of suicidal thoughts and behaviors linked to antidepressant therapy in pediatric patients [72]. Meta-analyses of placebo-controlled studies have indicated that antidepressants may cause a significant, although small and short-term, risk of self-harm or suicide-related events in children and adolescents with MDD, no completed suicides were reported in any trial included in the analysis [73,74]. Li et al. [75] reported that a history of attempted suicide was shown to be associated with a low level of antioxidant vitamins and carotenoids. Therefore, increasing plasma vitamin C levels in children and adolescents who are being treated with antidepressants may help mitigate some of this risk. However, as suicidal thoughts and behaviors were not measured in the present study, future clinical research is needed to test this hypothesis.

### Limitations

The present study has several limitations, one being its small sample size. While pilot clinical trials can play an important role in the early assessment of novel treatment methods when they are well designed and evaluated [76], further studies with larger sample sizes are needed to substantiate the results of this study. Secondly, drawing conclusions from a combined sample of children and adolescents with regard to the response to medication should be done with precaution as there is reason to believe that children respond differently than adolescents to antidepressants [77]. Also, due to the low potential for adverse drug reactions related to vitamin C in this study, the effect of doses higher than 1000 mg/day should be considered in future studies.

Measuring plasma vitamin C levels pre- and post-treatment may also be of interest, but although these levels were not measured, previous studies have demonstrated the association between hypovitaminosis C (vitamin C deficiency) and psychological abnormalities and this deficiency is highly prevalent in acutely hospitalized patients [32,36]. Furthermore, the increase in plasma and mononuclear leukocyte vitamin C from subnormal to normal concentrations after the administration of vitamin C administration implicate that the metabolic properties of hypovitaminosis C are consistent with deficiency as opposed to different mechanisms such as tissue redistribution [36]. These findings also indicate that patients with depression, such as those who participated in this study, may experience vitamin C deficiency and that the decrease in depressive symptoms that was observed may be directly attributed to the synergistic antidepressant effect of vitamin C and fluoxetine. Future studies that involve measuring plasma vitamin C levels may further support these findings. Finally, in the current study, participants were only treated and assessed for a short period of time (six months). The most striking effects were observed for the interaction between treatment and time and this finding suggests that longer trials are needed to better assess the efficacy of vitamin C as an adjunct to fluoxetine therapy.

## Conclusion

Treatment with 1000 mg/day of vitamin C potentiated the efficacy of fluoxetine in pediatric patients being treated for MDD. Furthermore, vitamin C was shown to be a particularly attractive therapeutic adjuvant due to the absence of substantial side effects and its inexpensive cost. The observed improvements in CDRS and CDI scores also imply that this type of treatment effectively increases blood plasma levels of vitamin C as it has been shown that ascorbic acid deficiency is associated with psychological abnormalities [26-29]. Future, large-scale clinical trials are warranted to evaluate the therapeutic efficacy of vitamin C for the treatment of depression in pediatric patients as well as its effectiveness as an adjuvant treatment to antidepressants.

## Competing interests

The authors declare that they have no competing interests.

## Authors’ contributions

All authors participated in the preparation of the manuscript and approved the final manuscript.
